# Cases of two patients with aortoduodenal fistula who underwent emergency operation

**DOI:** 10.1016/j.ijscr.2020.03.036

**Published:** 2020-04-01

**Authors:** Shinichiro Makimoto, Tomoya Takami, Hiroshi Shintani, Naoki Kataoka, Tomoyuki Yamaguchi, Masafumi Tomita, Yoshiharu Shono, Satoshi Kuroyanagi

**Affiliations:** a Department of Surgery, Kishiwada Tokushukai Hospital, 4-27-1, Kamori-cho, Kishiwada City, Osaka, 596-8522, Japan; b Department of Cardiovascular Surgery, Kishiwada Tokushukai Hospital, 4-27-1, Kamori-cho, Kishiwada City, Osaka, 596-8522, Japan

**Keywords:** Primary aortoduodenal fistula, Secondary aortoduodenal fistula, Endovascular aneurysm repair, Prosthetic graft replacement, Gastrointestinal bleeding

## Abstract

**Introduction:**

Aortoduodenal fistula (ADF) is a rare but life-threating condition, and it is an important cause of massive gastrointestinal bleeding. Primary ADF often occurs as a result of aortic aneurysm, and secondary ADF develops after the placement of aortic prosthetic graft.

**Presentation of cases:**

Case 1: A 64-year-old man with hematemesis was transferred to our hospital. The patient was diagnosed with primary ADF. Thus, we performed prosthetic graft replacement for an aortic aneurysm, and suturing of the duodenal fistula and duodenojejunal side-to-side anastomosis were performed. He was then discharged on the 35th postoperative day. After 2 years and 10 months, he died of other diseases. Case 2: A 76-year-old man with a history of abdominal aortic aneurysm repair with a prosthetic graft 5 years back who presented with hematemesis and melena was transferred to our hospital. The patient was diagnosed with secondary ADF, and an emergency endovascular aneurysm repair (EVAR) and suturing of the duodenal fistula were perfomed. He was transferred for rehabilitation purposes on the 108th postoperative day but eventually died of pneumonia 6 months after surgery.

**Discussion:**

ADF is associated with high mortality. Initial bleeding is usually minor and often intermittent. However, it leads to severe bleeding and hemorrhagic shock. EVAR is preferred over open surgery because it can be performed faster and is less invasive. Recently, in case of hemorrhagic shock, EVAR is used as first-line treatment.

**Conclusion:**

Accurate diagnosis and immediate treatment are important for the survival of patients with ADF.

## Highlights

• Aortoduodenal fistula is a rare cause of gastrointestinal bleeding. However, it is life threatening.
• Diagnosis requires a high level of clinical suspicion, and surgery can offer the best chance of survival.
• Primary aortoduodenal fistula is often caused by aortic aneurysm without any previous vascular intervention.
• Secondary aortoduodenal fistula occurs after surgical treatment for abdominal aortic aneurysm.

## 1 Introduction

Aortoduodenal fistula (ADF) is a rare disease and is a cause of gastrointestinal hemorrhage, which is a life-threating condition. It requires urgent diagnosis, emergency operation, and sufficient surveillance to reduce re-infection [1,2]. Along with the wide use of stent grafts, endovascular aneurysm repair (EVAR) has been attempted as first-line treatment for hemorrhagic shock [3]. Herein, we report a patient with primary ADF who underwent prosthetic graft replacement and another patient with secondary ADF who had EVAR.

This work has been reported in line with the SCARE criteria [4].

## 2 Presentation of cases

### 2.1 Case 1

**Fig. 1:**
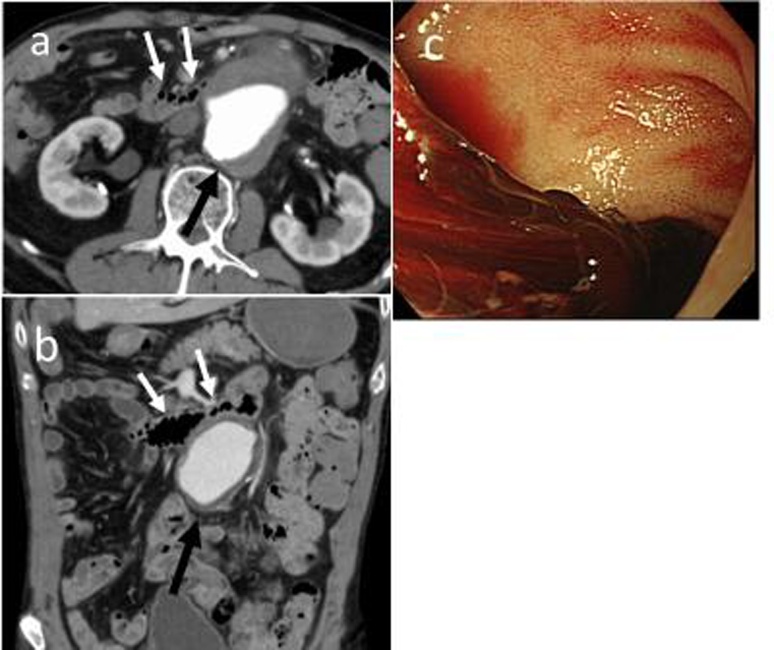
Abdominal computed tomography (CT) scan and upper gastrointestinal endoscopy (UGE). Axial (a) and coronal (b) abdominal CT scan revealed that the abdominal aortic aneurysm (black arrow) below the renal artery was dilated 7.6 × 5.4 cm, which compressed the duodenum (white arrows). (c) UGE revealed a large amount of fresh blood and blood clots in the bulb of the duodenum.

A 64-year-old man with hematemesis was transferred to the emergency unit of our hospital. The patient had a history of coronary artery bypass graft (CABG) and implantation of a pacemaker. Upper gastrointestinal endoscopy was performed. However, the source of bleeding was not clearly identified. Abdominal computed tomography (CT) scan revealed that the abdominal aorta below the renal artery was dilated by 7.6 × 5.4 cm (Fig. 1a and b); thus, imminent rupture was suspected. We then considered performing a scheduled surgery. However, the patient had massive hematemesis again after 11 days. Upper gastrointestinal endoscopy revealed a large amount of fresh blood and blood clots in the bulbs of the duodenum, and bleeding from the duodenum was suspected (Fig. 1c). Based on the laboratory examination result, the Hb level of the patient decreased to 8.9 g/dL. The patient lost consciousness, and he underwent emergency operation and was then diagnosed with ADF.

**Fig. 2:**
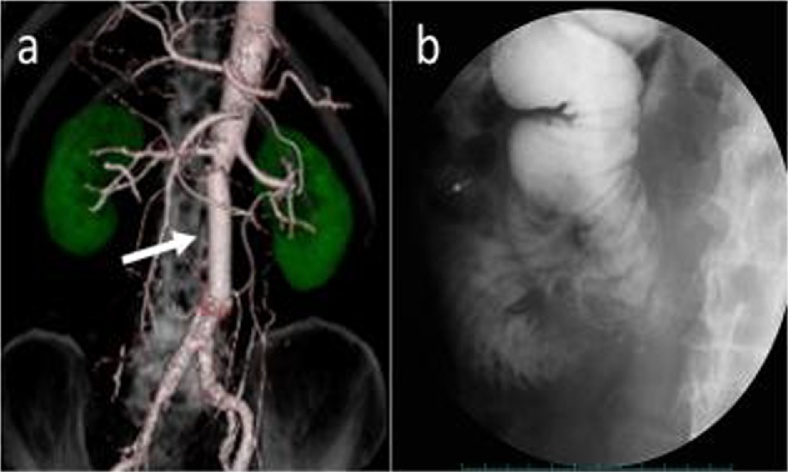
Abdominal computed tomography (CT) scan and duodenography. (a) Postoperative abdominal CT scan revealed that the I-shaped graft had good patency (white arrow). (b) Contrast media passed through the duodenojejunostomy without any problem.

During laparotomy, after proximal and distal aortic vascular control, the adhesion between duodenum and aortic aneurysm was released. Aortic aneurysm was replaced with prosthetic graft (Gelweave), which was covered with the omentum closely. The duodenal fistula (3 mm in diameter) was closed. The duodenum was resected on the anal side of the fistula, and side-to-side duodenojejunostomy was performed. He was discharged on the 35th postoperative day (Fig. 2a and b). However, he died of other diseases after 2 years and 10 months.

### 2.2 Case 2

**Fig. 3:**
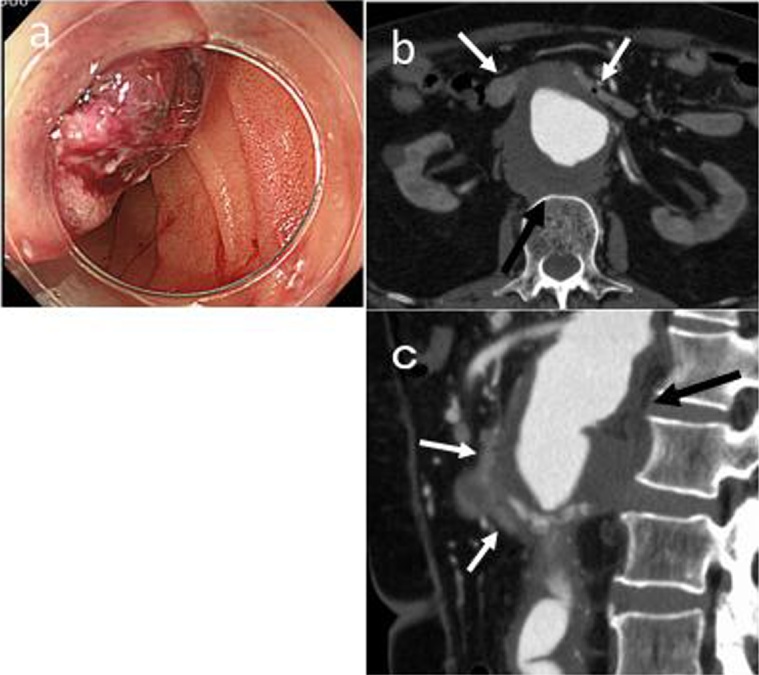
Upper gastrointestinal endoscopy (UGE)　and abdominal computed tomography (CT) scan. (a) UGE revealed the beating hematoma on the anal side of the major papilla. Axial (b) and sagittal (c) abdominal CT scan revealed the dilated irregularly shaped aneurysm (black arrow) on the proximal side of the prosthetic vascular graft, which compressed the duodenum (white arrows).

A 76-year-old man with hematemesis and melena was transferred to the emergency unit of our hospital. He had a history of prosthetic graft replacement for an abdominal aortic aneurysm (AAA) 5 years back, as well as open stent graft for arch aortic aneurysm and CABG 4 years back. Laboratory examination revealed that the patient had anemia (Hb level, 8.2 g/dL), chronic renal failure (Cr level, 2.84 mg/dL) and hyperkalemia (K level, 6.0 mEq/dL). Upper gastrointestinal endoscopy revealed a hematoma which pulsed on the anal side of the duodenal papilla (Fig. 3a). Abdominal CT scan revealed a dilated irregularly shaped aneurysm, which was located proximal to the aortic graft (Fig. 3b and c).　In addition, stenosis of the bilateral renal arteries was observed, and an aneurysm was also noted above the renal arteries; thus, it was strongly suspected that AAA penetrated the duodenum.

**Fig. 4:**
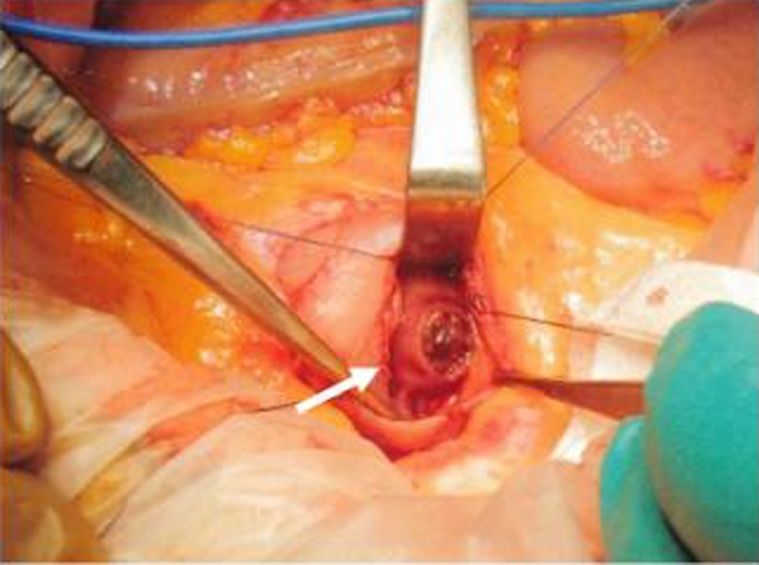
Operative finding. A 1-cm perforation was found in the third part of the duodenum (white arrow).

**Fig. 5:**
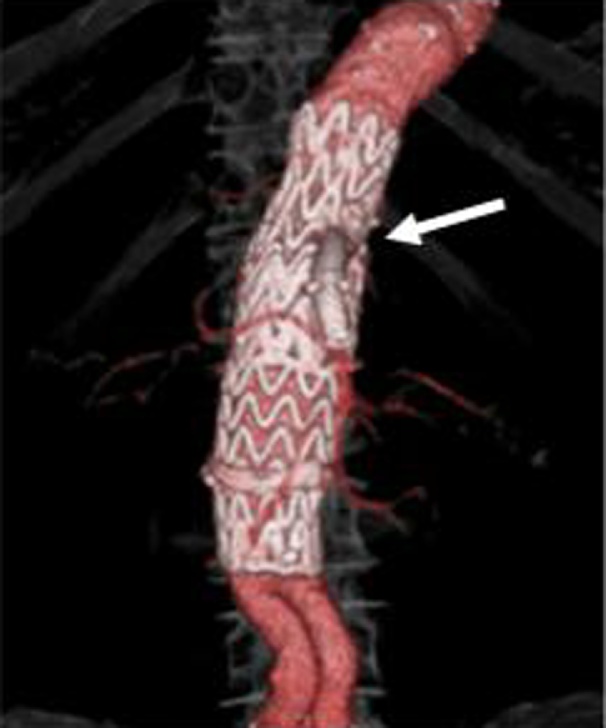
Abdominal computed tomography (CT) scan. Postoperative abdominal CT scan showed that endovascular aneurysm repair was performed and the superior mesenteric artery was reconstructed (white arrow).

The patient presented with hemorrhagic shock, and emergency endovascular aneurysmal repair (EVAR), celiac artery coil embolization, and superior mesenteric artery reconstruction (chimney method) were performed. Then, during laparotomy, adhesion between the third part of the duodenum and AAA was strong, and it was difficult to release. No obvious infection was observed around the fistula. We then incised the third part of the duodenum and closed the 1-cm fistula (Fig. 4). The patient underwent dialysis after surgery, and he was weaned from the ventilator on the 7th postoperative day (Fig. 5). The patient took a long term to improve postoperative respiratory function. Then, the patient was discharged on the 108th postoperative day and was transferred for rehabilitation purposes. He was re-hospitalized for pneumonia and respiratory failure after 2 months. However, he died 6 months after the operation.

## 3 Discussion

ADF is one of the aortic intestinal fistulas, and it occupies 80% of the aortic intestinal fistula [5,6]. It is classified into the two types. The first type is primary ADF, which is caused by arteriosclerotic aneurysm, infection, trauma, radiation therapy, and inflammatory aneurysm. This disease is extremely rare, with an incidence rate of 0.04%–0.07% during autopsy [5,7]．The　second type is secondary ADF, which is caused by prior aortic surgery with the replacement of prosthetic graft and is more common, with a post-operative incidence of 0.5%–2.3% [7].

In case 1, the patient presented with primary ADF, and in case 2, the patient had secondary ADF．In both cases, after the cardiovascular surgeons performed aortic aneurysm surgery, the gastrointestinal surgeons subsequently performed duodenal surgery.

The most common symptoms of ADF are hematemesis, melena, and abdominal pain [3]. Initial bleeding is usually minor and often intermittent. However, it leads to severe bleeding and hemorrhagic shock. Endoscopy is initially performed. However, the site of bleeding is sometimes difficult to identify if spontaneous hemostasis occurs [[7], [8], [9]]. Contrast CT scan is considered useful for diagnosis, with a sensitivity of 50–94% and a specificity of 85–100% [2].

When a patient is diagnosed with ADF, emergency operation is required. Traditionally, ADF was treated with extra-anatomic bypass graft and aortic ligation to prevent the use of a prosthetic graft at the infected site [3]. However, this technique was associated with an operative mortality rate of 25%–90% and an amputation rate of 5%–25% [3,10]. The long-term patency of the graft was recently identified in in situ aortic graft replacement, and it has been considered an alternative to ligation and bypass grafting [3]. The mean perioperative mortality rate for in situ reconstruction ranges from 27%–30% [3,11]．

EVAR has fewer complications and lower mortality and is preferred for high-risk patients or those on bridging therapy [12]. Recently, EVAR is selected for patients with hemorrhagic shock due to its hemostatic effect [3,12]. However, monotherapy with EVAR still can not eliminate the risk of graft infection at a later stage. The risk of re-infection remains high in patients with secondary ADF who undergo EVAR, and long-term administration of antibiotics is associated with a reduced re-infection rate [3]. EVAR should be considered a bridging procedure to definite open surgery if possible [2,3,[12], [13], [14]].

For duodenal repair, fistula treatment at an early stage is preferred. It is necessary to select an appropriate procedure based on site, size, surrounding infection, and extent of inflammation of the fistula. If the defect of the duodenal wall is about 1 cm to one-third of the circumference, simple closure or wedge resection of the wall can be performed, and when it is larger, partial resection or duodenal reconstruction is required [15,16].

In case 1, the duodenal fistula was sutured. The duodenum was resected on the anal side of the fistula; then, side-to-side duodenojejunostomy was performed. In case 2, no obvious infection around the fistula was observed. Adhesion around the aortoduodenal fistula was extremely strong; thus, we only closed the duodenal fistula. The patient had a risk of infection at a later stage, and he then received long-term antibiotic treatment.

The therapeutic approach for ADF is open surgery or EVAR and is mainly based on the state of hemorrhagic shock or not. EVAR should be performed as a bridging therapy, followed by definite open surgery [2]. However, if it is difficult to perform, the defect in the duodenum must still be repaired to prevent further contamination from duodenal contents [1,17].

## 4 Conclusion

Accurate diagnosis and immediate treatment are important for survival in patients with ADF. In situ aortic reconstruction has been performed as an alternative to aortic ligation and bypass grafting. Furthermore, EVAR is selected for patients with hemorrhagic shock due to its hemostatic effect or those on bridging therapy. Thus, it may be an important therapeutic strategy. In addition, reducing the risk of infection during the early stage of ADF treatment is important.

## Conflicts of interest

The authors declare no conflicts of interest.

## Sources of funding

This research did not receive any specific grant from funding agencies in the public, commercial, or not-for-profit sectors.

## Ethical approval

Ethical approval of this study was not required by our ethics committee.

## Consent

Written informed consent was obtained from both patients for publication of this case report and accompanying images.

## Author contribution

Makimoto S., Tomita M., Shono Y., and Kuroyanagi S. designed this report. Takami T., Shintani H., Kataoka N., and Yamaguchi T. contributed to the collection of data. Makimoto S. drafted the manuscript.

## Registration of research studies

This study was performed in accordance with the Declaration of Helsinki 2013. We registered at researchregistry. Our UIN is researchregistry5267.

## Guarantor

Shinichiro Makimoto.

## Provenance and peer review

Not commissioned, externally peer-reviewed.
